# Topology-Optimized Splints vs Casts for Distal Radius Fractures: A Randomized Clinical Trial

**DOI:** 10.1001/jamanetworkopen.2023.54359

**Published:** 2024-02-02

**Authors:** Honghong Ma, Beite Ruan, Jian Li, Jiahui Zhang, Changgui Wu, Hao Tian, Yichen Zhao, Debing Feng, Wei Yan, Xiaobing Xi

**Affiliations:** 1 Department of Orthopaedics, Shanghai Key Laboratory for Prevention and Treatment of Bone and Joint Diseases, Shanghai Institute of Traumatology and Orthopaedics, Ruijin Hospital, Shanghai Jiao Tong University School of Medicine, Shanghai, China; 2 Graduate School, Shanghai University of Traditional Chinese Medicine, Shanghai, China; 3 Clinical Research Center, Ruijin Hospital, Shanghai Jiao Tong University School of Medicine, Shanghai, China

## Abstract

**Importance:**

To date, there is currently no evidence-based medical support for the efficacy of topology-optimized splints in treating distal radius fractures.

**Objective:**

To assess the clinical efficacy and complication rates of topology-optimized splints in the treatment of distal radius fractures after closed manual reduction.

**Design, Setting, and Participants:**

This 12-week, multicenter, open-label, analyst-blinded randomized clinical trial (comprising a 6-week intervention followed by a 6-week observational phase) was carried out from December 3, 2021, to March 10, 2023, among 110 participants with distal radius fractures. Statistical analysis was performed on an intention-to-treat basis between June 3 and 30, 2023.

**Intervention:**

Participants were randomly assigned to 2 groups: the intervention group received topology-optimized splint immobilization and the control group received cast immobilization after closed manual reduction for 6weeks. After this period, immobilization was removed, and wrist rehabilitation activities commenced.

**Main Outcomes and Measures:**

The primary outcome was the Gartland-Werley (G-W) wrist score at 6 weeks (where higher scores indicate more severe wrist dysfunction). Secondary outcomes encompassed radiographic parameters, visual analog scale scores, swelling degree grade, complication rates, and 3 dimensions of G-W wrist scores.

**Results:**

A total of 110 patients (mean [SD] age, 64.1 [12.7] years; 89 women [81%]) enrolled in the clinical trial, and complete outcome measurements were obtained for 101 patients (92%). Median G-W scores at 6 weeks were 15 (IQR, 13-18) for the splint group and 17 (IQR, 13-18) for the cast group (mean difference, −2.0 [95% CI, −3.4 to −0.6]; *P* = .03), indicating a statistically significant advantage for the splint group. At 12 weeks, no clinically significant differences in G-W scores between the 2 groups were observed. Complication rates, including shoulder-elbow pain and dysfunction and skin irritation, were less common in the splint group (shoulder-elbow pain and dysfunction: risk ratio, 0.28 [95% CI, 0.08-0.93]; *P* = .03; skin irritation: risk ratio, 0.30 [95% CI, 0.10-0.89]; *P* = .02).

**Conclusions and Relevance:**

Findings of this randomized clinical trial suggest that patients with distal radius fractures that were managed with topology-optimized splints had better wrist functional outcomes and fewer complications at 6 weeks compared with those who received casting, with no difference at week 12. Therefore, topology-optimized splints with improved performance have the potential to be an advisable approach in the management of distal radius fractures.

**Trial Registration:**

Chinese Clinical Trial Registry: ChiCTR2000036480

## Key Points

**Question:**

Is a topology-optimized splint more effective than traditional cast therapy in reducing wrist pain and enhancing wrist function for patients with distal radius fractures, including those who did and did not undergo closed manual reduction?

**Findings:**

In this randomized clinical trial involving 110 participants with distal radius fractures, the intervention group received topology-optimized splint immobilization and the control group received cast immobilization after closed manual reduction for 6 weeks. The topology-optimized splint group exhibited better wrist functional outcomes than the traditional cast group for the treatment of distal radius fractures, with no clinically significant difference at 12 weeks of follow-up.

**Meaning:**

The results suggest that the topology-optimized splint provides early relief from pain and improved function for patients, offering effective treatment while mitigating the risks associated with surgery.

## Teaser

This randomized clinical trial assesses the clinical efficacy and complication rates of topology-optimized splints in the treatment of distal radius fractures after closed manual reduction.

## Introduction

Distal radius fractures (DRFs) are among the most common fracture injuries in emergency trauma departments, accounting for approximately 20% of all emergency fractures and 75% of forearm fractures.^1^ Predominantly resulting from indirect injuries such as falls, sports accidents, or traffic accidents,^2,3^ DRFs affect both young individuals and those older than 65 years. Younger patients typically experience high-energy injuries, whereas older adults experience low-energy incidents.^4,5^ The population of older adults in China is projected to exceed 200 million by 2025.^6^ Osteoporotic fractures, with DRFs being prominent among older adults, are becoming a major public health concern.^7^

Treatments for DRFs comprise surgical and conservative approaches. Several trials have shown that surgical treatment effectively achieves anatomical reduction and improves early mobility and quality of life vs conservative treatment.^8,9,10^ However, follow-up studies found no significant differences in pain or functional improvement between these approaches,^11,12,13,14,15^ sparking debate on the best strategy.^16^ British guidelines recommend closed reduction and cast immobilization as primary treatments.^17^ However, drawbacks of the conventional cast, such as bulkiness, have spurred treatment innovations.^18,19,20,21^ The rise of 3-dimensional (3D) printing in orthopedics has led to bespoke splints with superior clinical efficacy, lightweight design, and better ventilation.^22,23,24^ These splints could reduce patient pain, lessen risks of joint stiffness, and hasten wrist recovery.^25^

Topology optimization from engineering^26^ is used to make orthotic devices lighter. It mathematically models optimal structures and materials, maximizing strength and minimizing material use. This approach is beneficial, exemplified by Liao et al,^27^ who reduced a brace’s weight by 12.4% without efficacy loss. Similarly, Mian et al^28^ and Yan et al^29^ demonstrated topology-optimized splints’ potential in enhancing patient comfort and reducing splint weight. Despite advances, clinical studies on topology-optimized splints remain scarce. Recognizing this, we used topology optimization with 3D printing to develop a novel polyamide^30,31^ splint. We conducted preliminary safety evaluations of our splint using finite element simulations.

Given the absence of clinical trials on splints designed via topology optimization, our study initiates a 12-week randomized clinical trial. We aim to compare outcomes (Gartland-Werley [G-W] scores, radiographic parameters, visual analog scale [VAS] scores, swelling grade, complications) between patients treated with topology-optimized splints and those treated with conventional casts over 6 weeks. Our hypothesis posits that topology-optimized splints will be more effective than traditional casts in enhancing function, alleviating pain, and minimizing complications.

## Methods

### Study Design

The clinical trial protocol (Supplement 1) was approved by the ethics committee of Ruijin Hospital Affiliated to Shanghai Jiao Tong University School of Medicine and registered in the Chinese Clinical Trial Registry (2020 Clinical Ethics Review [238]). During the eligibility screening of patients with DRFs in the outpatient and emergency department of Shanghai Ruijin Hospital and Shanghai Yangpu District Hospital of Traditional Chinese Medicine from December 3, 2021 to March 10, 2023, all participants provided written informed consent. This study followed the Consolidated Standards of Reporting Trials (CONSORT) reporting^32^ reporting guideline, and the CONSORT flowchart is shown in the Figure.

**Figure.**
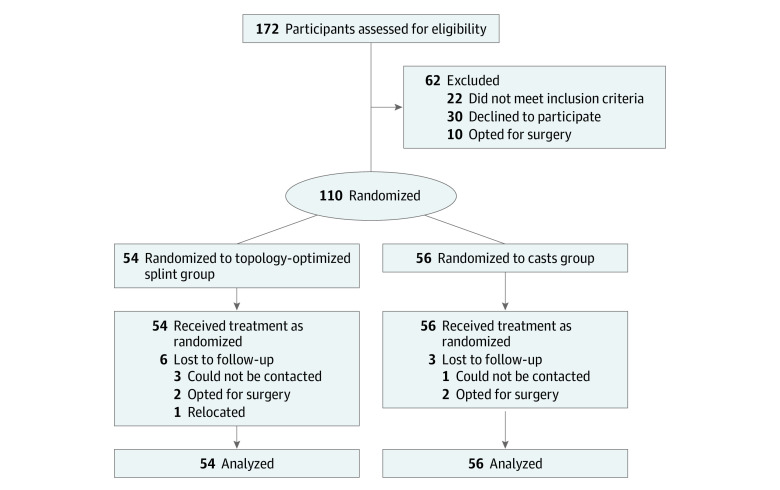
Study Flowchart

### Eligibility Criteria

In this study, potential participants with a diagnosis of DRF who met the following criteria were eligible: acute DRF, age 18 to 85 years, DRFs classified as type A and type B fractures according to AO Foundation/Orthopaedic Trauma Association (AO/OTA) classification,^33^ fractures both with and without displacement, displaced fractures requiring closed manual reduction, fresh closed fractures that have not been combined with other parts of the fracture and have not received other treatment methods, willing to sign informed consent, and willing to cooperate with the physician for voluntary follow-up. Exclusion criteria were as follows: acute open fracture or pathological fracture; inability to achieve functional reduction after closed reduction of fracture (radiographic criteria after reduction^34^: dorsal angulation >10°, radial inclination <15°, or radial shortening >3 mm); rheumatic diseases requiring hormone therapy for >6 months; serious cardiovascular and cerebrovascular diseases, diabetes, or neurologic or psychiatric diseases; and wrist skin damage, infection, and ulceration.

### Randomization, Allocation Concealment, and Blinding

Randomization was implemented by an independent statistician. A stratified block randomization was applied and stratified by center. A block randomization was applied within each center. The block size in our study was accessible only to the statistician who performed the randomization and generated the allocation sequence. Patients were randomly assigned to the topology-optimized splint group or the cast group in a 1:1 ratio. Random sequence lists generated by SAS, version 9.4 (SAS Institute Inc), were sealed in opaque envelopes and distributed to each center. The envelope was opened in front of the patient in chronological order of the patient’s visit. The statisticians and data analysts were blinded to the group assignments, which was implemented to ensure the impartiality of the analysis. Patients were screened and followed up by surgeons and clinical research coordinators (CRCs) to whom the interventions were clearly visible. Surgeons and CRCs were not involved in the data analysis process.

### Interventions

All surgeons involved in this study were trained in the treatment of DRFs and the application of splints and casts. In accordance with routine clinical practice, the fracture was reduced by closed manipulation under local anesthesia (nondisplaced fractures do not require closed reduction). Radiographic films were used to assess the success of reduction after closed manual reduction (eAppendix 3 in Supplement 2). External immobilization was achieved using splints and casts, according to the assigned group. After immobilization, participants were encouraged to engage in finger activity.

In the cast group, the cast was applied directly over the skin to immobilize the fracture, extending from below the elbow to the metacarpals. Conversely, in the splint group, prefabricated topology-optimized splints (eAppendix 3 in Supplement 2), which were crafted by combining topology optimization technology with 3D printing technology, were used to immobilize the fracture. For patients presenting unique anatomical considerations, customized topology-optimized splints were made available. The detailed technical methods for both the splints and casts are described in eAppendix 2 in Supplement 2. Swelling degree and pain were observed during follow-up appointments at 3 days, 1 week, and 2 weeks. If severe swelling and discomfort were present, adjustments to the splint or cast may have been necessary. External immobilization was typically removed after a mean (SD) of 6.0 (0.2) weeks for both groups. Participants in both groups were provided with the same detailed home exercise program (eAppendix 1 in Supplement 2), and any additional rehabilitation exercise (including physical therapy) beyond the program was at the discretion of the clinician or patient.

### Outcome Measures

Participants were followed up and data were collected by the CRCs at 3 days, 1 week, 2 weeks, 6 weeks, and 12 weeks. Baseline data included age, sex, fracture type (AO [Arbeitsgemeinschaft für Osteosynthesefragen] classification), fractured wrist, VAS score, grading of swelling degree, radiographic parameters of fracture, radiographic parameters after reduction of fracture, and comorbidities.

The primary outcome was the G-W wrist score (range, 0-38; higher scores indicate worse pain) evaluated at 6 weeks. This score integrates 4 key dimensions: residual deformities (presence of residual radial or dorsal deformity), subjective evaluation (patient’s self-assessment of pain, limited mobility, and loss of function), objective evaluation (surgeon’s assessment of wrist range of motion, distal radioulnar joint pain, and grip strength), and complications (arthritis, neuropathy, and poor finger function). In addition, we examined 3 dimensions of the G-W score as secondary outcomes to thoroughly understand patients’ recovery trajectories under different treatment modalities.

Secondary outcomes included changes in fracture displacement (radius height, volar angle, or radius inclination) and fracture healing at 2, 6, and 12 weeks after reduction using radiographs; VAS score changes at 3 days, 7 days, 2 weeks, 6 weeks, and 12 weeks (range, 0-10 points, with 0 indicating no pain and 10 indicating the most severe pain); degree of swelling in the wrist joint at 3 days, 7 days, 2 weeks, 6 weeks, and 12 weeks (with 0 degrees indicating no swelling, 1 degree indicating mild swelling with visible skin lines, 2 degrees indicating obvious skin swelling with disappearing lines, and 3 degrees indicating skin depression and tension blisters); and complications such as arthritis, complex regional pain syndrome, carpal tunnel syndrome, shoulder-elbow pain and dysfunction, tension blisters, skin irritation syndrome (redness, swelling, itching, allergies, dermatitis, and odor), and poor finger function.

### Statistical Analysis

Statistical analysis was performed between June 3 and 30, 2023. The sample size was calculated using PASS software, version 11 (NCSS), with reference to a previous randomized clinical trial (n = 94)^35^ that compared splint and cast treatments for DRFs. The findings revealed that after 3 months, the splint group had an rate of 96% on the G-W score, while the cast group had a rate of 77%. Assuming a 19% difference in G-W score between the 2 groups, with a 2-sided 5% probability of type I error and 80% power, the sample size was determined to be 100 patients (50 per group). Considering a potential dropout rate of 10%, a total of 110 patients were ultimately required for enrollment.

Data analysis was consistent with the principle of intention-to-treat, with data incorporated from all enrolled participants. To address the issue of missing data, we used multiple imputation methods to estimate both primary and secondary outcomes for all follow-up periods. Considering the nonmonotonic nature of the missing data, we used the fully conditional specification method within the canonical multiple imputation framework. Continuous data that followed a normal distribution were presented as mean (SD), while data that did not follow a normal distribution were presented as median (IQR). Disaggregated data were expressed as a percentage. Comparisons between the groups used the *t* test or the Mann-Whitney test for continuous variables and the χ^2^ test or Fisher exact test for categorical variables. For repeatedly measured data, a linear mixture model was used for statistical analysis, with participants as random effects, and time and group as fixed effects. Multiple comparisons of repeatedly measured data were Bonferroni corrected. Complications were analyzed by calculating the risk ratios and 95% CIs. All tests were 2-sided, and *P* < .05 was considered statistically significant. SPSS software, version 24.0 (IBM Corp), was used for all statistical analyses.

## Results

### Participant Characteristics

From December 3, 2021, to March 10, 2023, a total of 172 eligible patients were screened and 110 participants (mean [SD] age, 64.1 [12.7] years; 89 women [81%]) were recruited, with 54 assigned to the splint group and 56 to the cast group. Nine participants withdrew from the study. In the splint group, 6 participants dropped out for the following reasons: 2 chose surgery, 1 relocated, and 3 could not be contacted. In the cast group, 3 participants withdrew: 2 opted for surgery, while 1 was lost during follow-up. Ultimately, 101 participants completed the study (Figure). The baseline characteristics of the participants were found to be similar in both the splint and cast groups (Table 1). Throughout the clinical trial, the surgeon and the CRC consistently supervised and managed the follow-up of the patients.

**Table 1.** Baseline Characteristics of Participants

Characteristic	Splint group (n = 54)	Cast group (n = 56)
Age, median (IQR) [range], y	67 (59 to 70) [24 to 85]	66 (57 to 72) [29 to 85]
Sex, No. (%)		
Male	13 (24)	8 (14)
Female	41 (75)	48 (86)
Fractured wrist, No. (%)		
Left wrist	27 (50)	31 (55)
Right wrist	27 (50)	25 (45)
AO classification, No. (%)^a^		
A type	38 (70)	35 (63)
B type	16 (30)	21 (38)
VAS score, median (IQR)^a^	6 (4 to 7)	6 (4 to 7)
Grading of swelling degree, median (IQR)^b^	1 (1 to 2)	1 (1 to 2)
Radiographic parameters of fracture, median (IQR)^c^		
Radial height, mm	9 (8 to 11)	9 (7 to 11)
Volar angulation, °	3 (−10 to 10)	2 (−11 to 10)
Radial inclination, °	20.5 (17 to 23)	20 (15 to 23)
Reduction of fracture, median (IQR)		
Radial height, mm	10 (9 to 11)	10 (9 to 11)
Volar angulation, °	7 (4 to 11)	8 (4 to 11)
Radial inclination, °	22 (20 to 23)	21 (19 to 24)
Comorbidities, No. (%)		
Diabetes	12 (22)	7 (13)
Smoking	6 (11)	5 (9)

Abbreviations: AO, Arbeitsgemeinschaft für Osteosynthesefragen class; VAS, visual analog scale.

^a^ On the VAS score, higher scores indicate worse pain.

^b^ On the grading of swelling degree, higher scores indicate worse pain.

^c^ Measured by computer imaging system.

### Participant-Reported Outcomes

Table 2 presents a detailed overview of the G-W scores. After 6 weeks, the splint group had a significantly lower overall median G-W score compared with the cast group (15 [IQR, 13-18] vs 17 [IQR, 13-18]; mean difference, −2.0 [95% CI, −3.4 to −0.6]; *P* = .03). The splint group also reported a lower subjective score than the cast group. Both groups showed limited wrist range of motion, but the splint group had advantages in dorsal flexions, with a mean difference of 10.1 (95% CI, 5.2-14.9; *P* < .001). Furthermore, the splint group experienced a lower incidence of distal radioulnar joint pain compared with the cast group (35 of 48 [73%] vs 47 of 53 [89%]; *P* = .04). At 12 weeks, after 6 weeks of rehabilitation training, both groups demonstrated significant improvements in wrist range of motion and grip strength, reaching approximately 70% of the strength of their uninjured hand. However, wrist functions had not yet returned to preinjury levels, and no significant differences were observed between the 2 groups.

**Table 2.** Primary Outcomes at 6 and 12 Weeks

Clinical outcome	At 6 wk		Splint vs cast, mean difference (95% CI)	*P* value	At 12 wk		Splint vs cast, mean difference (95% CI)	*P* value
	Splint group (n = 54)	Cast group (n = 56)			Splint group (n = 54)	Cast group (n = 56)		
G-W score, median (IQR), points^a^	15 (13 to 18)	17 (13 to 18)	−2.0 (−3.4 to −0.6)	.03	0.5 (0 to 2)	0 (0 to 2)	−0.05 (−0.6 to 0.5)	.84
Residual deformity, No. (%)^b^	2 (4)	3 (6)	NA	>.99	NA	NA	NA	NA
Subjective rating, No. (%)^c^								
Optimal	1 (2)	1 (2)	NA	.03	37 (69)	41 (73)	NA	.59
Good	1 (2)	3 (5)			17 (31)	15 (27)		
Average	30 (56)	16 (29)			0	0		
Poor	22 (40)	36 (64)			0	0		
Objective evaluation, median (IQR), % of uninjured side^d^^,^^e^								
Dorsal flexion, °	30 (24 to 45), 41^f^	22 (18 to 30), 30	10.1 (5.2 to 14.9)	<.001	70 (65 to 74), 84	70 (63 to 75), 84	0.6 (−1.5 to 2.7)	.49
Volar flexion, °	31 (24 to 38), 40	32 (21 to 40), 39	0.7 (−3.7 to 5.2)	.74	68 (60 to 75), 82	65 (60 to 70), 81	0.9 (−1.7 to 3.5)	.54
Pronation, °	72 (62 to 81), 85	69.5 (58 to 78), 84	1.3 (−2.3 to 4.8)	.45	85 (80 to 85), 99	85 (80 to 85), 99	0.1 (−0.9 to 1.1)	.61
Supination, °	49 (43 to 52), 66	48 (44 to 50), 65	1.2 (−0.5 to 2.9)	.25	65 (60 to 69), 89	64.5 (60 to 70), 90	−0.2 (−2.2 to 1.8)	.86
Radial deviation, °	6 (4 to 9), 30	6 (5 to 8), 31	−0.1 (−1.0 to 0.8)	.66	20 (18 to 20), 93	19.5 (17 to 20), 91	0.4 (−0.2 to 1.0)	.33
Ulnar deviation, °	20 (17 to 22), 65	20 (18 to 21), 64	0.1 (−1.3 to 1.5)	.74	28 (27 to 28), 91	27 (27 to 28), 91	0.08 (−0.4 to 0.5)	.84
Grip strength, kg^g^	5 (4 to 7), 25	5 (3 to 7), 23	0.5 (−0.5 to 1.4)	.44	16 (12 to 18), 73	15 (13 to 17), 71	0.8 (−0.9 to 2.4)	.52
Ring motion defect, No. (%)	46 (85)	49 (88)	NA	.72	8 (15)	7 (13)	NA	.72
Distal radioulnar joint pain, No. (%)	39 (76)	50 (89)	NA	.02	9 (17)	12 (21)	NA	.53

Abbreviations: G-W, Gartland-Werley; NA, not applicable.

^a^ G-W score range, 0 to 38; higher scores indicate worse pain. The G-W score was the main results and all other results were secondary.

^b^ Ulnar styloid protrusion, residual radial or dorsal deformity (according to imaging assessment).

^c^ Assessment by the patient of pain, limitation of movement, or loss of function.

^d^ Measure by electronic protractor.

^e^ Percentage compared with uninjured side.

^f^ Mean of percentage.

^g^ Measure by electronic grip strengthener.

Radiographic parameters, VAS scores, and wrist swelling levels were compared between groups (Table 3). Throughout 12 weeks of follow-up, no noticeable differences in radiographic parameters after closed reduction were observed between the 2 groups, although both groups exhibited varying degrees of fracture displacements. Analysis of VAS score changes from baseline to 12 weeks revealed that the splint group experienced more significant pain reduction compared with the cast group at both the 2- and 6-week intervals. Specifically, the adjusted mean difference in VAS score from baseline reduction favored the splint group by −0.52 (95% CI, −1.05 to 0.02; *P* = .03) at 2 weeks and −0.55 (95% CI, −1.09 to −0.01; *P* = .03) at 6 weeks. At 2 weeks, the splint group (–0.26; 95% CI, −0.48 to −0.05, *P* = .02) also showcased superior reductions in swelling compared with the cast group.

**Table 3.** Secondary Outcomes

Radiographic parameter^a^	Median (IQR)		Mean change from reduction baseline (95% CI)		Splint vs cast, mean difference (95% CI)	*P* value
	Splint group (n = 54)	Cast group (n = 56)	Splint group (n = 54)	Cast group (n = 56)		
**Radial height, mm^b^**						
Reduction baseline	10 (9 to 11)	10 (9 to 11)	NA	NA	NA	NA
At 2 wk	10 (9 to 11)	10 (9 to 11)	–0.13 (–0.26 to –0.04)	–0.23 (–0.41 to –0.07)	0.10 (−0.10 to 0.31)	.40
At 6 wk	9.5 (9 to 11)	10 (8 to 11)	–0.24 (−0.41 to –0.09)	–0.48 (–0.73 to –0.27)	0.24 (−0.05 to 0.53)	.12
At 12 wk	9.5 (9 to 11)	10 (8 to 11)	–0.26 (0.43 to –0.11)	–0.48 (0.73 to –0.25)	0.22 (−0.07 to 0.51)	.18
**Volar angulation, °** ^c^						
Reduction baseline	7 (4 to 11)	8 (4 to 11)	NA	NA	NA	NA
At 2 wk	7 (3.5 to 11)	7.5 (4 to 11)	–0.22 (−0.44 to –0.04)	–0.73 (–1.59 to –0.11)	0.51 (−0.27 to 1.29)	.95
At 6 wk	7 (2.75 to 11)	7.5 (4 to 11)	–0.57 (–0.91 to –0.28)	–0.93 (–1.77 to –0.34)	0.35 (−0.45 to 1.16)	.63
At 12 wk	7 (3.5 to 11)	7.5 (4 to 11)	–0.46 (–0.80 to –0.17)	–0.94 (–1.75 to –0.32)	0.47 (−0.34 to 1.27)	.78
**Radial inclination, °** ^d^						
Reduction baseline	22 (20 to 24)	21 (19 to 24)	NA	NA	NA	NA
At 2 wk	21 (19 to 24)	21 (18 to 24)	–0.52 (–1.03 to –0.13)	–0.79 (–1.23 to –0.38)	0.26 (−0.38 to 0.91)	.19
At 6 wk	21 (19 to 23)	21 (17 to 23.75)	–0.72 (–1.24 to –0.31)	–1.05 (–1.54 to –0.57)	0.33 (−0.36 to 1.02)	.16
At 12 wk	21 (19 to 23)	21 (17 to 23.75)	–0.80 (–1.30 to –0.39)	−1.05 (−1.55 to −0.57)	0.26 (−0.42 to 0.94)	.42
**VAS scores** ^e^ ^,^ ^f^						
Baseline	6 (4 to 7)	6 (4 to 7)	NA	NA	NA	NA
At 3 d	5 (3 to 6)	5 (3 to 6)	–0.98 (–1.31 to –0.67)	–0.89 (–1.18 to –0.61)	0.09 (−0.52 to 0.34)	.92
At 7 d	4 (3 to 5)	4 (3 to 4.75)	–2.06 (–2.41 to –1.72)	–2.09 (–2.39 to –1.80)	0.03 (−0.42 to 0.49)	.83
At 2 wk	2 (2 to 3)	3 (2 to 4)	–3.44 (–3.83 to –3.07)	–2.93 (–3.27 to –2.61)	–0.52 (–1.05 to 0.02)	.03
At 6 wk	1 (1 to 2)	2 (1 to 3)	–4.43 (–4.81 to –4.04)	–3.88 (–4.21 to –3.54)	–0.55 (–1.09 to –0.01)	.03
At 12 wk	0 (0 to 1)	0 (0 to 1)	–5.35 (–5.74 to –4.94)	–5.30 (–5.73 to –4.88)	–0.05 (−0.65 to 0.55)	.84
**Grading of swelling degree, %** ^g^ ^,^ ^h^						
Baseline	1 (1 to 2)	1 (1 to 2)	NA	NA	NA	NA
At 3 d	1 (1 to 2)	1 (1 to 2)	0.24 (0.13 to 0.37)	0.34 (−0.21 to 0.50)	–0.10 (−0.28 to 0.08)	.24
At 7 d	1 (1 to 2)	1 (1 to 2)	−0.02 (−0.19 to 0.15)	0.05 (−0.05 to 0.16)	–0.07 (−0.27 to 0.13)	.57
At 2 wk	1 (0 to 1)	1 (1 to 2)	–0.48 (–0.65 to –0.33)	–0.22 (–0.36 to 0.07)	–0.26 (–0.48 to –0.05)	.02
At 6 wk	0 (0 to 1)	1 (0 to 1)	–0.81 (–0.98 to –0.65)	–0.66 (–0.82 to 0.50)	–0.15 (−0.39 to 0.08)	.19
At 12 wk	0 (0 to 0)	0 (0 to 0)	–1.15 (–1.31 to –1.00)	–1.20 (–1.36 to –1.04)	0.05 (−0.28 to 0.19)	.75

Abbreviations: NA, not applicable; VAS, visual analog scale.

^a^ Measured by computer imaging system.

^b^ Radial height, mm: group × time interaction risk ratio is 1.08 (95% CI, 1.01-1.16); group risk ratio is 1.01 (95% CI, 0.52-1.96); time risk ratio is 0.84 (95% CI, 0.80-0.89).

^c^ Volar angulation,°: group × time interaction risk ratio is 1.13 (95% CI, 0.94-1.37); group risk ratio is 0.63 (95% CI, 0.05-8.33); time risk ratio is 0.74 (95% CI, 0.65-0.85).

^d^ Radial inclination,°: group × time interaction risk ratio is 1.09 (95% CI, 0.93-1.27); group risk ratio is 1.14 (95% CI, 0.28-4.66); time risk ratio is 0.71 (95% CI, 0.63-0.79).

^e^ On the VAS score, higher scores indicate worse pain.

^f^ VAS scores: group × time interaction risk ratio is 0.94 (95% CI, 0.87-1.01); group risk ratio is 1.05 (95% CI, 0.66-1.66); time risk ratio is 0.35 (95% CI, 0.34-0.37).

^g^ On the grading of swelling degree, higher scores indicate worse pain.

^h^ Grading of swelling degree, %: group × time interaction risk ratio is 1.00 (95% CI, 0.96-1.04); group risk ratio is 0.85 (95% CI, 0.69-1.06); time risk ratio is 0.77 (95% CI, 0.75-0.79).

### Complications

Table 4 provides an overview of the adverse events reported by both groups. These events encompassed arthritis, complex regional pain syndrome, carpal tunnel syndrome, tension blister, and finger dysfunction. However, there was no statistically significant difference observed between the groups in relation to these complications. The splint group exhibited a lower occurrence of shoulder-elbow pain and dysfunction (risk ratio, 0.28 [95% CI, 0.08-0.93]; *P* = .03) as well as skin irritation (risk ratio, 0.30 [95% CI, 0.10-0.89]; *P* = .02) compared with the cast group.

**Table 4.** Adverse Events

Adverse event	Splint group, No. (%) (n = 54)	Cast group, No. (%) (n = 56)	Risk ratio (95% CI)^a^	*P* value
Arthritis	9 (17)	10 (18)	0.92 (0.34-2.48)	.87
Complex regional pain syndrome	2 (4)	5 (9)	0.39 (0.07-2.12)	.44
Carpal tunnel syndrome	3 (6)	5 (9)	0.60 (0.14-2.64)	.72
Shoulder-elbow pain and dysfunction^b^	5 (9)	14 (25)	0.31 (0.10-0.94)	.03
Tension blister	5 (9)	7 (13)	0.71 (0.21-2.41)	.59
Skin irritation symptom^c^	5 (9)	15 (27)	0.28 (0.09-0.83)	.02
Poor finger function	7 (13)	9 (16)	0.78 (0.27-2.26)	.64

^a^ Risk ratios are expressed as splint vs cast.

^b^ The surgeon checks for pain and range of motion in the shoulder and elbow joints.

^c^ Redness, swelling, itching, allergies, dermatitis, and odor on the skin.

## Discussion

In this randomized clinical trial, most patients saw positive outcomes, effective treatment, reduced disability, and enhanced functional activity, leading to an elevated quality of life. At 6 weeks, G-W scores were more favorable among the splint group than the cast group. Participants who received topology-optimized splints reported better ability and satisfaction with daily living, but by 3 months, any differences had disappeared and all participants showed satisfactory results. Topology-optimized splints demonstrated advantages in alleviating wrist pain, enhancing functional activity, and boosting patient comfort in early period. Although both groups exhibited some fracture displacement after closed reduction, it may have been due to the limitations of external immobilization methods.^36,37^ Similar results have been demonstrated by other researchers. For displaced fractures, palmar plate fixation has been found to provide better stability and radiologic results compared with cast immobilization for DRFs. Unfortunately, these issues cannot be addressed with current conservative treatment. However, recent studies^38^ have introduced a new angle, suggesting that for older patients with DRFs, functional outcomes are not significantly correlated with the radiographic measures of reduction. This insight highlights that factors beyond exact wrist anatomical alignment play a role in realizing satisfactory outcomes. It broadens our approach to treatment strategies and emphasizes the importance of focusing on overall patient functionality and quality of life. The splint group outperformed the cast group in reducing VAS scores at 2 and 6 weeks, as well as in reducing wrist swelling at 2 weeks. Rehabilitation showed improvement in complications such as intra-articular fracture–induced arthritis. The cast group reported more shoulder-elbow complications, which could be attributed to adverse shoulder-elbow motion after cast immobilization, resulting in joint adhesion, pain, and dysfunction. Complications were less common among the splint group. In addition, the cast group experienced more skin irritation symptoms, possibly due to the lack of ventilation provided by the cast. In contrast, the ventilated structure of the splint led to fewer such symptoms among the splint group.

Traditional casts, often used for DRFs, are prone to complications such as arthritis, carpal tunnel syndrome, and skin issues,^21,39^ mainly from uneven pressure, excessive strength, and poor ventilation. 3D printing technology offers personalized, rapid solutions. Chen et al^40,41^ used this technology in designing external fixation tools for DRFs, incorporating perforations for enhanced ventilation, and subsequently conducted clinical trials for validation. The outcomes revealed that patients in the 3D printed cast group achieved superior therapeutic effects. Moreover, patient satisfaction surveys indicated a preference for the 3D printed casts compared with their traditional counterparts. Despite the evident clinical efficacy of the lightweight, breathable 3D printed casts, issues arose, including blisters near the ulnar bone and perforated skin indentations. Various researchers have opted for a uniform distribution of perforations,^42,43^ but this design has occasionally compromised fracture stability, caused skin to be pinched by the holes,^40^ and resulted in pressure ulcers due to high pressure points.^44^

This research used topology optimization technology in splint design,^29^ where the pressure distribution across the forearm was gauged using sensors. The resultant design ensured a balanced pressure distribution, optimizing structural integrity and material usage. The biocompatible material, polyamide—compliant with United States Pharmacopeia and National Formulary class I to VI and US Food and Drug Administration guidelines for skin surface devices,^45^ and noted for its rigidity and pliability^30,31^—was used in the 3D printing process, making it a popular choice in medical application.^46,47^ In addition, the inclusion of a porous sponge within the splint aids in preventing skin indentations and augments patient comfort.

Currently, there is a paucity of data regarding the treatment of DRFs using topology-optimized splints. This study introduces the use of 3D printed topology-optimized splints in the clinical realm of DRFs. Our findings underscore the potential of topology-optimized splints in effectively managing DRFs, marking a novel approach to external fixation techniques.

### Limitations

Our study has some limitations. First, both the surgeon and CRCs were exposed to treatment groups, eliminating blinding. Second, we excluded C3 fractures (AO/OTA classification), deeming them unsuitable for nonsurgical treatment, which limits the generalizability of our results. Third, while we excluded patients with certain chronic conditions, we acknowledge the potential benefits of nonoperative treatments. Future studies may focus on the specific patient population to explore the efficacy and safety of nonoperative methods in managing DRFs. Furthermore, the follow-up period in our study extended to 12 weeks, which may not have been sufficient to observe long-term results. We plan to continue tracking patient outcomes over an extended period to evaluate treatment efficacy and identify any long-term disparities more effectively. Fourth, the sample size was a study constraint.

## Conclusions

In this randomized clinical trial, patients with DRFs who received topology optimized splints showed greater improvements functional activity, pain, swelling, and complication rates than those who received casts in the early period. These findings indicate that topologically optimized splints are appropriate for the treatment of DRFs. Future larger multicenter randomized clinical trials are warranted.

## Notes

Supplement 1.
Trial Protocol

Supplement 2. **eAppendix 1.** Rehabilitation Exercise Program **eAppendix 2.** Splint and Cast Technique Methods **eAppendix 3.** Clinical Photos and Radiographs

Supplement 3.
Data Sharing Statement
