# Supplementary material for: Topology-Optimized Splints vs Casts for Distal Radius Fractures: A Randomized Clinical Trial
Source: JAMA Netw Open. 2024 Feb 2;7(2):e2354359. doi: 10.1001/jamanetworkopen.2023.54359 (PMC10837751; doi:10.1001/jamanetworkopen.2023.54359)
Supplement: Supplement 1. — Trial Protocol [file jamanetwopen-e2354359-s001.pdf]

Study Protocol

Clinical Research Program,

Ruijin Hospital, Shanghai Jiao Tong University

Study Name	Effectiveness of topologically optimized splint for distal radius fractures: A Randomized Clinical Trial
Program Number	SHDC2020CR3016A
Principal Investigator	Prof. Xiaobing Xi
Group leader unit	Ruijin Hospital, Shanghai Jiaotong University School of Medicine
Program Start Date	2021.12
Program End Date	2022.6

Catalog .... Error! Bookmark not defined.
1 Research Summary 4
1.1Abstract 4
1.2Schematic diagram of the study 5
2 Research Background 5
2.1 Research significance 5
2.2 Research Background 5
2.3 Expected results of the study 6
2.4 Potential risk/benefit assessment 6
2.4.1 Known potential risks 6
2.4.2 Known potential benefits 6
2.4.3 Potential risk/benefit assessment 6
3 Principal Investigator Information 7
3.1 Principal investigator's name, qualifications, contact information 7
3.2 Key participants 7
4 Purpose of the study 7
Main purpose 7
Secondary purpose 7
5 Study Design 7
5.1 Overall design 7
5.2 Study endpoints 7
5.3 Sample size 7
6 Research Subjects 7
6.1 Eligibility Criteria 8
6.2 Exclusion Criteria 8
6.3 Study Subject Recruitment 8
6.4 Methodology of research subject assignment 8
7 Research interventions 8
7.1 Intervention content 8
7.1.1 Study intervention description 8
7.1.2 Dosing and Administration 9
7.1.3The items and number of clinical and laboratory tests to be performed 9
7.2 Preparation/Handling/Storage/Accountability 9
7.2.1 Accountability 9
7.2.2 Formulation, appearance, packaging and labeling 9
7.2.3 Product storage and stability 9
7.2.4 Preparation 9
7.3 Measures to minimize bias 9
7.4 Follow-up and Adherence 9
7.5 Research Intervention Commitment 10
7.6 Research Flow Chart 10
8 Study intervention termination and subject termination/withdrawal 10
8.1 Study intervention termination 10
8.2 Subject termination/withdrawal 10

8.3 Lost to follow-up 11
9 Evaluation of study outcomes 11
9.1 Primary and secondary efficacy evaluation indicators 11
9.2 Safety evaluation indicators 11
9.3 Adverse Events and Serious Adverse Events 11
9.3.1 Adverse Event (AE) Definition 11
9.3.2 Serious Adverse Event (SAE) Definition 11
9.3.3 Adverse Event Reporting 11
9.3.4 Serious Adverse Event Reporting 12
10 Statistical Analysis 12
10.1 General Method 12
10.2 Analysis of primary and secondary study endpoints 12
10.3 Baseline descriptive analysis 13
11 Supporting Documents and Notes 13
11.1 Informed Consent 13
11.2 Privacy Protection 13
11.3 Specimen and data collection and use 13
11.4 Experimental Process Management 13
11.5 Data processing and record keeping 14
11.5.1 Data Collection and Management 14
11.5.2 Research data retention 15
11.6 Publishing and data sharing conventions 15
11.7 Conflict of interest statement 15
12 References 15

1 Research Summary

1.1 Abstract

**Study Name**	A randomized clinical study of topology-optimized splint in the treatment of distal radius fractures
**Research Introduction**	This is a multicenter, randomized controlled clinical study with plans to enroll patients with distal radius fractures. Eligible participants were randomly assigned 1:1 to the intervention group and the control group to be immobilized with a topology-optimized splint or cast, respectively. Participants' fractured wrist were collected before and during the intervention.
**Purpose of research**	To assess the clinical efficacy and complication rates of topology-optimized splints in the treatment of distal radius fractures following closed manual reduction.
**Research subjects**	Meet the diagnostic criteria for distal radius fracture, aged between 18 and 85 years. It is planned to include 110 participants.
**Research Unit**	Principal Investigator and Affiliation: Prof. Xiaobing Xi, Ruijin Hospital, Shanghai Jiaotong University School of Medicine
**Research interventions**	Intervention group: Topology-optimized splint immobilization for 6 weeks, rehabilitation exercise for 6 weeks, 3 days, 7 days, 2 weeks, 6 weeks and 12 weeks of observation follow-up.<br>Control group: Cast immobilization for 6 weeks, rehabilitation exercise for 6 weeks, 3 days, 7 days, 2 weeks, 6 weeks and 12 weeks of observation follow-up.<br>Evaluation metrics： Gartland-Werley (G-W) wrist scores, radiographic parameters, visual analogue scale (VAS) scores, swelling degree grade, complication rates
**Duration of research**	Start in 12,2021,expected to be completed in 6 2023
**Subjects' participation time**	12weeks

1.2 Schematic diagram of the study

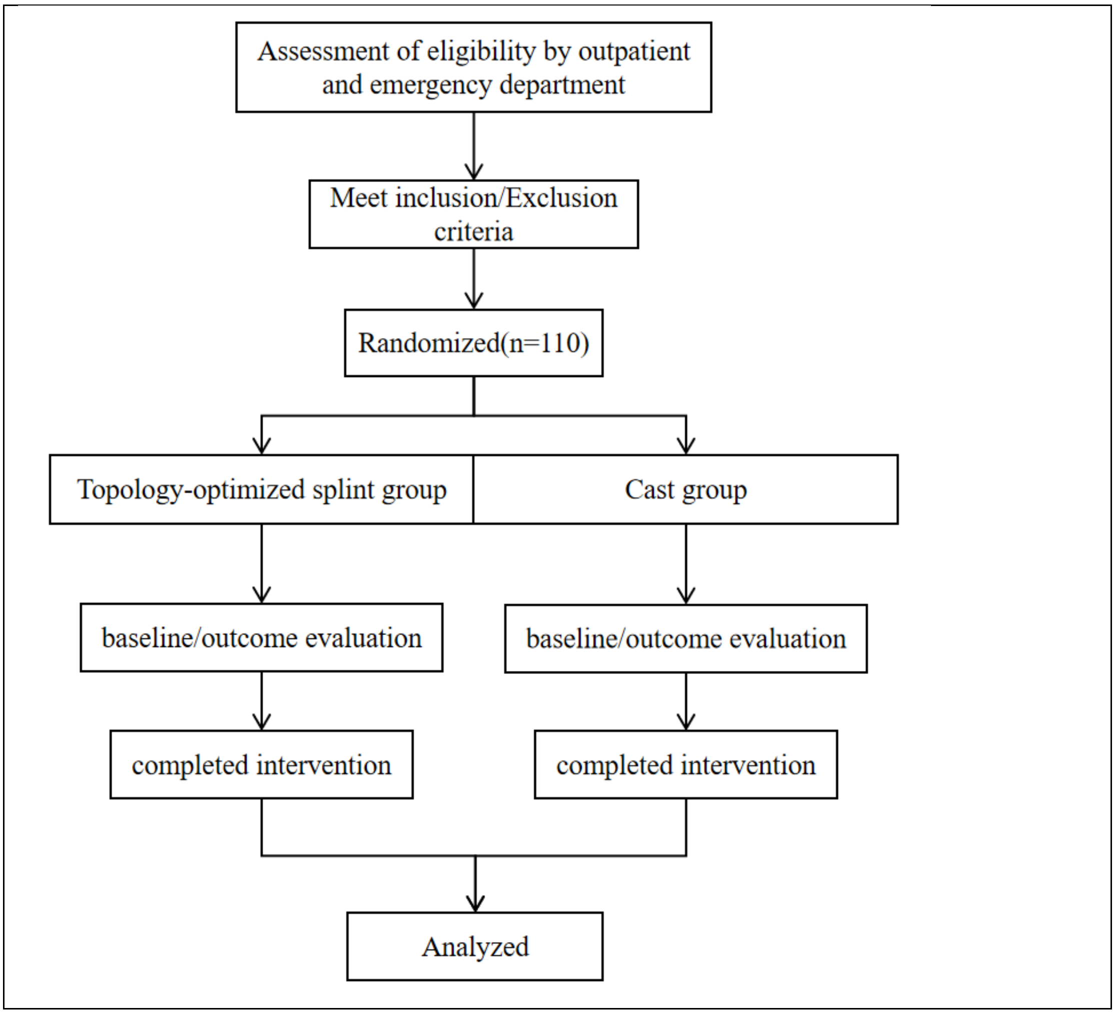

2 Research Background

2.1 Research significance

Distal radius fractures are a serious public health problem in the world. As the global population ages, osteoporosis and the risk of falls increases, the incidence of distal radius fractures is increasing year by year. From the perspective of patients' wishes and medical insurance, the elderly are more suitable for conservative treatment. Compared with the elderly, the lightweight and breathable external fixation method is more suitable for them. We use topology optimization technology to design the splint to ensure structural strength and improve comfort. Safety tests were carried out using finite element simulation analysis in the early stage, but clinical studies have not yet been carried out. A randomized controlled trial was conducted to verify the efficacy of topology-optimized splints for distal radius fractures.

2.2 Research Background

Distal radius fractures (DRFs) represent one of the most frequent fracture injuries encountered in emergency trauma departments. Accounting for approximately 20% of all emergency fractures and 75% of forearm fractures[1], their prevalence is undeniable. Predominantly resulting from indirect violent injuries like falls with an outstretched hand, sports or traffic accidents[2,3], DRFs have a broader reach, commonly affecting both young individuals and those over 65. While younger patients often suffer from high-energy injuries, the elderly tend to experience DRFs due to low-energy incidents[4,5]. Notably, as the elderly population in China is projected to surpass 200 million by 2025[6]. osteoporotic fractures, with

DRFs as the second most common among the elderly, are emerging as significant public health concerns [7].

DRFs treatments are dichotomized into surgical and conservative approaches. Interestingly, 12 or 24-month follow-up studies reveal no pronounced difference in pain or functional improvement between these two treatments[8-12], fueling debates over the optimal strategy[13]. British guidelines advocate for closed reduction and cast immobilization as primary treatment modalities[14]. However, the conventional cast's drawbacks, like bulkiness and discomfort, have led to innovations in treatment options[15-18]. With the rise of 3D printing in orthopedics, bespoke splints boasting superior clinical efficacy, lightweight design, and enhanced ventilation are emerging[19-21]. Such splints could potentially ease patient pain, diminish joint stiffness risks from prolonged immobilization, and expedite wrist function recovery[22].

The concept of topology optimization, originally from engineering[23], is being employed to make orthotic devices more lightweight. By mathematically modeling the optimal structural and material distributions for given conditions, the method maximizes strength while minimizing material use. This approach has proven beneficial. For instance, Liao et al[24] successfully reduced the Boston brace's weight by approximately 12.4% without compromising its corrective efficacy. Similarly, Mian et al[25] and Yan et al[26]showcased the potential of topology-optimized splints in enhancing comfort and reducing weight. However, despite these advances, clinical studies examining topology-optimized splints remain scarce. Recognizing this, we employed topology optimization coupled with 3D printing to craft a novel splint from polyamide (PA12)[27,28]. Preliminary safety evaluations of our topology-optimized splint have been conducted through finite element simulations

2.3 Expected results of the study

The purpose of this project was to verify the efficacy and safety of splinting for distal radius fractures. The splint combines engineering topology optimization technology and 3D printing technology to ensure the strength of the structure, improve comfort, and meet the needs of patients with distal radius fractures. Distal radius fracture splints can be used at different times of fracture, which can save a large number of patients with distal radius fractures from surgery, reduce medical costs, and facilitate community promotion.

2.4 Potential risk/benefit assessment

2.4.1 Known potential risks

All randomized patients may encounter the risks of anesthesia. These risks include: allergic reactions, nerve damage, drug reaction, slowing or stopping of breathing, failure of the anesthetic or sedation analgesia, cardiac arrest, risks that cannot be predicted, permanent disability or even death.

All randomized patients may encounter the general risks of distal radius fracture. These include infection, pain, bleeding, deformity, nerve injury, tendon injury, decreased range of motion, malunion or nonunion, future arthritis (if fracture involves intra-articular) and the need for further surgery.

All patients may encounter the risk of loss of privacy or confidentiality. All research personnel involved in any way with this project will have completed training in the protection of human research participants.

2.4.2 Known potential benefits

All participants were provided with a professional treatment plan. Each subject will receive good results at the end of the trial.

2.4.3 Potential risk/benefit assessment

Significant fracture displacement and serious complications may occur in participants, but are rare clinically. During the trial, the patient should be fully informed of the precautions and the necessary

simple and operable treatment methods. In the study, the investigator will evaluate the safety of the subjects. Therefore, the potential risks of this study are controllable, and the potential benefits outweigh the potential risks

3 Principal Investigator Information

3.1 Principal investigator's name, qualifications, contact information

Unit Ruijin Hospital, Shanghai Jiao Tong University

Name Prof. Xiaobing Xi

Phone 021-64370045-666096

3.2 Key participants

Serial number	Name	Gender	Age	Title	Specialties	Whether GCP training	Role in the study (eg: PI, sub-I, CRC)
1	Xi Xiaobing	Male	49	Chief physician	surgery	Yes	PI
2	Zhang Hao	Male	50	Deputy chief physician	surgery	Yes	Sub-I
3	Jia Youji	Male	34	Attending physician	surgery	Yes	Sub-I
4	Yan Wei	Male	28	Resident doctor	surgery	Yes	Sub-I
5	Ruan Beite	Male	25	Resident doctor	surgery	Yes	Sub-I
6	Ma Honghong	Male	26	Resident doctor	surgery	Yes	Sub-I

4 Purpose of the study

Main purpose: To assess the clinical efficacy of topology-optimized splints in the treatment of distal radius fractures following closed manual reduction.

Secondary purpose: To evaluate the effects of topology-optimized splints on radiographic parameters, pain, swelling, and complications of distal radius fractures.

5 Study Design

5.1 Overall design

The study was a multicenter, randomized, analyst-blind controlled trial. Each subject was randomly assigned to an intervention group or a control group in chronological order of visit. The intervention group was immobilization with topology-optimized splint, and the control group was immobilization with cast. After 6 weeks of immobilization, the patient was instructed to perform wrist rehabilitation exercises and the clinical outcomes were evaluated.

5.2 Study endpoints

Subjects are considered to have completed all studies if they have completed all phases of outcome assessment or follow-up according to the study protocol.

5.3 Sample size

The sample size was calculated by PASS software (NCSS, Kaysville, UT) with sample allocation ratio of 1:1 between 2 groups with reference to other studies (n=94)[29]. The difference in G-W score effective rate between the intervention group (96%) and the control group (77%) was 19% at 3 months, with a 2-sided 5% of type I error probability, and an 80% of power, allowing drop-out rate of 10%, a total of 110 cases were finally required for enrollment.

6 Research Subjects

6.1 Eligibility Criteria

In order to be eligible to participate in this study, an individual must meet all of the following criteria:

1.acute distal radius fracture

2.with men or women aged 18 to 85 years old

3.patients with DRFs were classified as type A and type B fractures according to AO/OTA classification

4.fresh closed fractures that have not been combined with other parts of the fracture and have not received other treatment methods

5.fresh closed fractures that have not been combined with other parts of the fracture and have not received other treatment method

6.participants who were willing to cooperate with the doctor for voluntary follow-up

6.2 Exclusion Criteria

An individual who meets any of the following criteria will be excluded from participation in this study:

1.patients with acute open fracture or pathological fracture

2.patients with bilateral DRFs

3.patients who cannot achieve functional reduction after closed reduction of fracture (radiographic criteria after reduction: dorsal angulation>10° , radial inclination <15° , or radial shortening >3 mm)

4.Patients with rheumatic disease who need to take hormone for a long time; patients with serious cardiovascular and cerebrovascular diseases, diabetes, neurological or psychiatric diseases

5. patients with wrist skin damage, infection and ulceration

6.patients who do not agree to be randomized

7.patients with current substance abuse

6.3 Study Subject Recruitment

Study subjects were recruited by surgeons and clinical coordinators (CRCs). During the eligibility screening of patients with distal radius fractures in the outpatient and emergency department of Shanghai Ruijin Hospital and Shanghai Yangpu District Hospital of Traditional Chinese Medicine. Subjects will be enrolled after signing an informed consent form, being diagnosed by surgeons and CRC, and being evaluated according to the study protocol. Planned recruitment options include, but are not limited to, newspapers, the Internet, and posters.

6.4 Methodology of research subject assignment

Patients were randomly assigned to the topologically optimized splint group or the cast group in 1:1 ratio. Random sequence list generated by SAS (Strategic Application Software, version 9.4; SAS Institute Inc) were sealed in opaque envelopes and distributed to each center. The envelope was opened in front of the patient in chronological order of the patient's visit.

7 Research interventions

7.1 Intervention content

7.1.1 Study intervention description

In accordance with routine clinical practice, the fracture was reduced by closed manipulation under local anesthesia. Imaging X-ray films were used to determine whether a satisfactory closed reduction was achieved after reduction. Splint and cast were used for external immobilization according to the group. Active finger activity was encouraged after immobilization. External immobilization was removed for an average of 6 weeks, and wrist rehabilitation activities commenced.

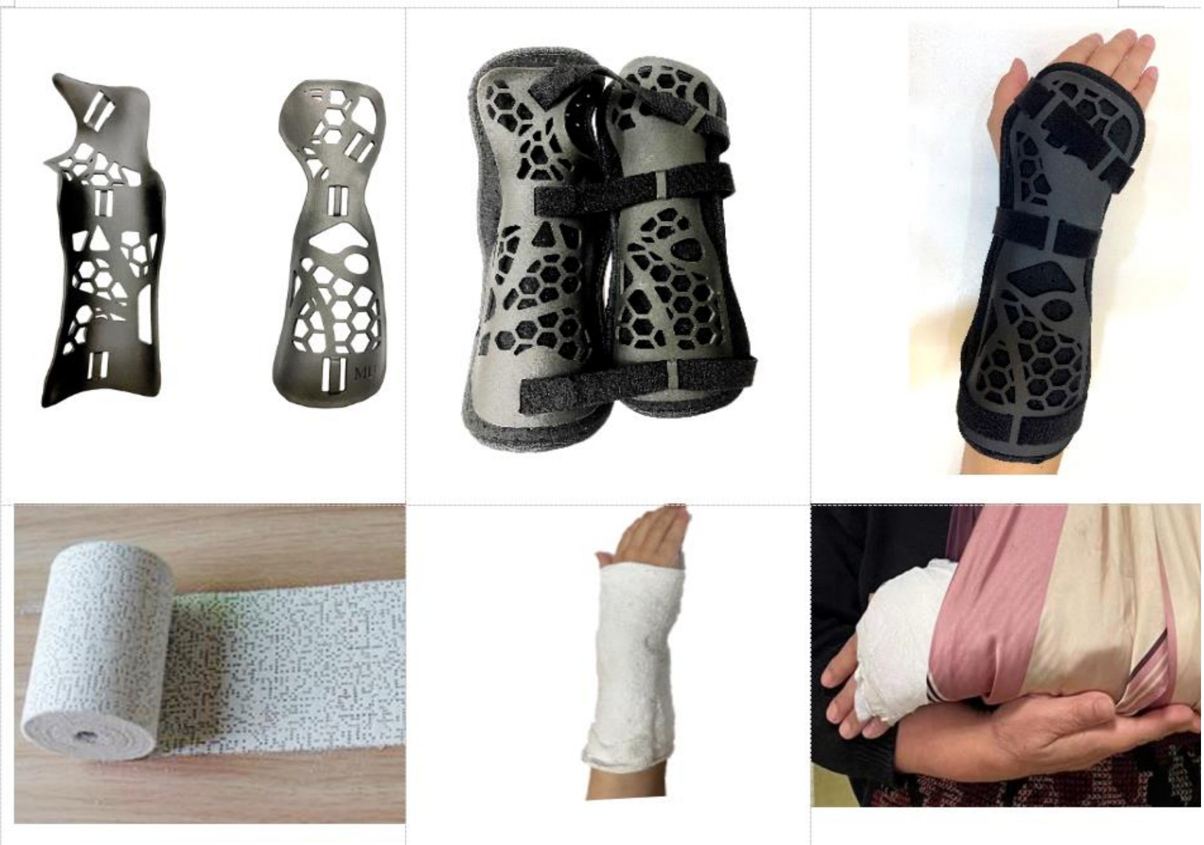

7.1.2 Dosing and Administration

Not applicable

7.1.3 The items and number of clinical and laboratory tests to be performed

The fractured wrist was X-rayed examinations four times after reduction, 2weeks, 6 weeks, and 12weeks, to assess displacement and healing

7.2 Preparation/Handling/Storage/Accountability

7.2.1 Accountability

The researcher should record the brand and type information of the splint and cast.

7.2.2 Formulation, appearance, packaging and labeling

Not applicable

7.2.3 Product storage and stability

Storage under normal conditions, no special requirements

7.2.4 Preparation

All surgeons involved in this study were trained in the treatment of distal radius fractures and the application of splints and casts. In accordance with routine clinical practice, the fracture was reduced by closed manipulation under local anesthesia. Splint and cast were used for external immobilization according to the group.

7.3 Measures to minimize bias

Randomization, Allocation Concealment, and Blinding

7.4 Follow-up and Adherence

The observation period was 3 months, and participants were evaluated clinically at 3 days, 7 days, 2 weeks, 6 weeks, and 12 weeks after initiation of treatment, with X-ray evaluation at 2 weeks, 6 weeks, and 12 weeks.

The researchers should carefully implement informed consent so that the subjects fully understand the test requirements and cooperate with the researchers. The surgeon treated the participants free of charge and directed rehabilitation training

7.5 Research Intervention Commitment

Participants were immobilized in topology-optimized splints and casts and recovered at the end of the study, while various situations that arose during the intervention were recorded and solutions were discussed in time

7.6 Research Flow Chart

	Screening Period	Treating Period				Follow-up Period
**Time**	**Day0**	**Day3**	**Day7**	**Week2**	**Week6**	**Week12**
**Basic information**						
Informed Consent Form	√					
Medical history, past medical history	√					
Inclusion/exclusion criteria	√					
Subjects were randomized	√					
**General evaluation and safety evaluation**						
visual analogue scale (VAS) scores	√	√	√	√	√	√
Grading of swelling degree	√	√	√	√	√	√
radiographic parameters	√			√	√	√
Gartland-Werley (G-W) wrist scores					√	√
Adverse Event Reporting		√	√	√	√	√
Summary of research completion						√
Drug combination		√	√	√	√	√
**Other work**						
Assign random numbers	√					
Distribution research instrument	√					
CRF filling	√	√	√	√	√	√

8 Study intervention termination and subject termination/withdrawal

8.1 Study intervention termination

During the course of the study, if significant fracture displacement or serious complications such as compartment syndrome occur, the intervention study should be stopped and immediate surgical treatment should be performed, and the available data should be recorded in detail.

After the study intervention was discontinued, the subjects continued to follow up regularly to observe the swelling, pain and movement of the affected limb, and to take reasonable measures.

The termination of the study intervention does not mean that the entire study is termination, and the remaining study procedures continue in accordance with the current treatment protocol for distal radius fractures.

8.2 Subject termination/withdrawal

The researcher can suspend or withdraw the research object when the research object has the following conditions:

(1) Pregnancy

(2) Significant research intervention noncompliance

(3) In the event of significant fracture displacement or other clinical conditions, it is no longer in the subjects' best interest to continue participating in the study

(4) The disease progresses to the point where discontinuation of the study intervention is required

Subjects' reasons for termination/withdrawal from the study should be recorded on the case report form, and subjects who signed informed consent and were randomly assigned but did not receive the study intervention will be replaced. Study subjects who sign informed consent, are randomly assigned, receive the study intervention and then withdraw will be or not replaced.

8.3 Lost to follow-up

Subjects who stop scheduled study follow-up, fail to complete prescribed study procedures, or cannot be contacted by the investigator may be considered lost to follow-up. Notify subjects in time window in advance to reduce lost follow-up and missing data.

Each subject should leave a detailed contact address, telephone number, ID card and other information, and the doctor should leave his or her contact number with the subject, so that the subject can seek the doctor's help when the condition changes, which is also conducive to the doctor to keep abreast of the condition changes, remind the subject to return visit in time, and avoid the loss of visit.

9 Evaluation of study outcomes

9.1 Primary and secondary efficacy evaluation indicators

9.2 Safety evaluation indicators

(1) physical examination: vital signs, Respiration, heart rate, blood pressure

(2) Severe arm pain, compartment syndrome

(3) Skin Adverse Reactions: erythema, itching, vesicles, exudation, pigmentation changes, desquamation, etc., at the local site

(4) Any adverse reactions that may occur during the trial and abnormal changes in related test parameters

(5) Adverse Events

9.3 Adverse Events and Serious Adverse Events

9.3.1 Adverse Event (AE) Definition

Adverse Events: The term “adverse events” encompasses the occurrence or worsening of any signs, symptoms, syndromes, or diseases that affect the health of subjects during the observation period of a clinical study. This term also includes clinically relevant situations discovered during laboratory or other diagnostic procedures, such as the need for unplanned medical intervention, withdrawal from the study, or abnormal laboratory test results. Adverse events may be: new diseases; worsening of symptoms, signs, or conditions related to the treatment; worsening of comorbidities; effects of control drugs; unrelated to participation in the study; or a combination of one or more factors. Therefore, the term "adverse events" does not necessarily imply a causal relationship with the study drug.

9.3.2 Serious Adverse Event (SAE) Definition

Serious Adverse Event (SAE): Refers to the following adverse events that occur at any dose of the investigational drug or at any time during the observation period, including death or life-threatening events, hospitalization or prolonged hospitalization, permanent disability, teratogenic effects, induction of cancer, events of significant medical importance (meaning those that do not immediately jeopardize life or lead to death or require hospitalization but may harm the patient or require measures to prevent one of the consequences defined above), necessitating medical intervention to prevent permanent damage or harm.

9.3.3 Adverse Event Reporting

All AEs (whether related to the experimental intervention or not) must be fully documented from the time the informed consent form is signed until the end of the study.

9.3.4 Serious Adverse Event Reporting

For all serious adverse events occurring during a clinical study, whether reported for the first time or as a follow-up, the investigator must immediately complete and sign and date a Serious Adverse Event Report Form and report it immediately (no later than 24 hours after the SAE is known) to the The applicant, the ethics Committee and the group leader, and written report to the local provincial drug supervision and administration department, Health Bureau adverse event detection event center. The investigator should ensure that all required information is submitted within the above time frame.

In general, a sufficiently detailed description of the adverse event should be included to allow for a thorough medical evaluation of the case and an independent assessment of the likelihood of causality. In addition, the investigator must provide information on other possible causes of the adverse event, such as combined medications and comorbidities. In the event of a subject's death, the investigator must submit a summary of autopsy results (if available) to the professional organization for adverse drug reaction monitoring of the province, autonomous region or municipality directly under the Central Government as soon as possible. contact information for SAE reporting is detailed in the investigator's file provided to each center. The original SAE report form must be kept in a safe place by the center.

10 Statistical Analysis

10.1 General Method

During statistical analysis, the following steps will be taken: first, an examination of the number of completed cases and dropout status of the patients; then, an analysis of the baseline characteristics related to demographics for the two groups at the time of patient inclusion, assessing comparability between the study group and the control group; efficacy evaluation will involve determining efficacy indicators and comparing the efficacy between the two groups; safety evaluation will include a comparison of laboratory indicators and clinical adverse reactions.

All statistical tests will be two-tailed, and a p-value less than or equal to 0.05 will be considered statistically significant for the tested differences.

Data analysis was consistent with the principle intention-to-treat (ITT) of per-protocol (PP).For quantitative variables, descriptive statistics will include means, standard deviations, medians, minimum values, maximum values, lower quartiles (Q1), upper quartiles (Q3), and for categorical variables, the description will include counts and percentages for each category.

Comparisons between the two groups for general characteristics will be analyzed using appropriate methods based on the type of indicator.The comparison between the two groups used Student's t-test or the Mann–Whitney U-test for continuous variables, and chi-square test or Fisher's exact test for categorical variables. For repeatedly measured data, we used a linear mixture model for statistical analysis, with participants as random effects, time and group as fixed effects, multiple comparisons of data were correction by Bonferroni. Complications were analyzed by calculating the risk ratios and 95% confidence interval.

10.2 Analysis of primary and secondary study endpoints

Analysis of primary study endpoints:

Gartland-Werley (G-W) score: The comparison between the two groups used Student' s t-test or the Mann–Whitney U-test for continuous variables, and chi-square test or Fisher' s exact test for categorical variables.

Analysis of secondary study endpoints:

Radiographic parameters: mixed linear model to test for differences between groups (participants as random effects, time and group as fixed effects).

visual analogue scale (VAS) score, swelling degree grade: mixed linear model to test for differences between groups (participants as random effects, time and group as fixed effects).

Complication: chi-square test or Fisher’s exact test for categorical variables, analyzed by calculating the risk ratios and 95% confidence interval.

10.3 Baseline descriptive analysis

Baseline information for both groups was recorded as descriptive statistics. Continuous variables are expressed as mean ± standard deviation, categorical variables are expressed as percentages, and skewed variables are expressed as medians (IQR).

11 Supporting Documents and Notes

11.1 Informed Consent

Informed consent should be obtained before research subjects agree to participate in the study and should be maintained throughout the entire research process. The informed consent document, approved by the ethics committee, should be provided to research subjects for their review. Researchers will explain the research process and answer any questions posed by research subjects, informing them of potential risks and their rights. Research subjects may discuss their participation with family members or guardians before consenting. Researchers must convey to research subjects that participation is voluntary and they can withdraw from the study at any time. Copies of the informed consent form will be provided to research subjects for their records. The rights and welfare of research subjects will be protected, emphasizing that the quality of their medical care will not be affected by their decision to decline participation in the study.

11.2 Privacy Protection

The researcher must maintain original data records for each patient. (Original data includes: an informed consent form signed by the patient with the trial number and trial name, X-ray examinations, etc.) Information appearing on the case report form should be traceable to these original data sources. Only members of the research team are authorized to access this information, and no research information can be disclosed to unauthorized third parties without the approval of the authorized national authority.

11.3 Specimen and data collection and use

The use and analysis of specimens and information materials involved in the study are conducted at Shanghai Ruijin Hospital, Shanghai Jiao Tong University School of Medicine. The responsible research unit has established a dedicated location within Ruijin Hospital for the storage of data, which is overseen by designated personnel. Additionally, in accordance with national regulations, basic information about the trial participants should be retained for a sufficient duration (typically 5 years after the trial concludes). At that time, the applicant will notify the researchers/institution that there is no need to further maintain records related to the trial. This study does not involve foreign background research institutions/companies**.**

11.4 Experimental Process Management

1.Investigator Qualifications: Researchers participating in clinical trials must undergo qualification review and possess the professional background and capabilities required for conducting clinical trials.

2.Laboratory Testing: Each participating hospital in the clinical trial will establish uniform standards and requirements for laboratory testing parameters.

3.Research Staff Training: Prior to the commencement of the clinical trial, research staff will undergo training to ensure a comprehensive understanding and awareness of the specific details and components of the clinical trial protocol.

4.Clinical Trial Monitoring: A monitoring officer will be appointed to conduct regular monitoring

visits to oversee the progress and completion of the trial. The monitoring officer will assess the completeness of case records, the accuracy of case report forms, verify trial data, examine compliance with the trial protocol and clinical trial management standards, and gather information on the progress of enrolled patients. Researchers and relevant personnel should assist the monitoring officer in their work and provide appropriate workspace for them.

11.5 Data processing and record keeping

11.5.1 Data Collection and Management

(1) Data Recording:

All cases must diligently complete the Case Report Forms (CRF) in accordance with this protocol. The attending physician should promptly fill out the CRF when the patient visits and ensure the accuracy of the data; all fields must be filled in, and no empty or omitted fields are allowed.

CRF serves as the original record, and any corrections can only be made by crossing out the error, noting the corrected data, explaining the reason, and having the participating clinical researcher sign and date it. Original records should not be erased or covered up.

Significant deviations from the normal range or data outside clinically acceptable limits (e.g., laboratory test results exceeding the normal range) should be verified, and necessary explanations should be provided by the participating clinical researcher.

Original lab reports should be attached to the CRF. The laboratory results should also be recorded in the relevant sections of the CRF, ensuring consistency with the data on the original lab report.

Data recording on the CRF: After the observation period for each subject concludes, the researcher should submit the CRF and informed consent form to the project leader for review and signature within 7 working days. They should also be archived in the institutional data archive room, with any issues discovered addressed and documented promptly.

(2) Data Monitoring:

The number of monitors and the frequency of visits should meet the quality control requirements of the clinical trial. Monitors should review each study case and CRF and complete the "Monitor's Review Page" for each case.

(3) Data Management:

Completion and Handover of Case Report Forms:

Researchers complete the Case Report Forms, with each enrolled case requiring a completed CRF. After clinical monitors review the completed CRFs, the first copy is handed over to the data manager for data entry and management.

Data Entry and Modification:

Data entry and management are the responsibility of the statistical unit's data manager. A dedicated database is established for data entry and management. To ensure data accuracy, two data managers should independently perform double data entry and verification.

For any questions in the CRF, data managers will fill out a Query Resolution Form (DRQ) and send inquiries to the researchers through clinical monitors. Researchers should promptly respond and return the answers. Data managers will make data modifications based on the researchers' responses, confirm, and enter the data. DRQs can be resent if necessary.

(4) Data Locking:

After data auditing and confirming the accuracy of the established database, the primary investigator, applicant, statistical analysts, and drug regulatory personnel will lock the data. Data files locked after this stage should not be altered. Any issues discovered after data locking can be corrected within the

statistical analysis program once confirmed.

11.5.2 Research data retention

The minimum retention period for all research data and original documents is 5 years after the conclusion of the study, and permission should be obtained before disposal.

11.6 Publishing and data sharing conventions

The data obtained in this project are owned by the project team and are generally shared among the project team members. Any publications or outcomes belong to the project team.

11.7 Conflict of interest statement

None of the investigators or research associates participating in this study had any personal financial or non-financial interests or any direct or indirect obligations or responsibilities that conflicted with their job duties during this study.

12 References

1. MacIntyre NJ, Dewan N. Epidemiology of distal radius fractures and factors predicting risk and prognosis. *J Hand Ther.* 2016;29(2):136-144.
2. Hippisley-Cox J, Coupland C. Derivation and validation of updated QFracture algorithm to predict risk of osteoporotic fracture in primary care in the United Kingdom: prospective open cohort study. *BMJ-British Medical Journal.* 2012;344:16.
3. Shehovych A, Salar O, Meyer CER, Ford DJ. Adult distal radius fractures classification systems: essential clinical knowledge or abstract memory testing? *Ann R Coll Surg Engl.* 2016;98(8):525-531.
4. Barrett JA, Baron JA, Karagas MR, Beach ML. Fracture risk in the US Medicare population. *J Clin Epidemiol.* 1999;52(3):243-249.
5. Chen NC, Jupiter JB. Management of distal radial fractures. *J Bone Joint Surg-Am Vol.* 2007;89A(9):2051-2062.
6. Mu HZ, Han ZB. China’s Economic Growth Expectation and Its Ｒealization Path in the Ｒapid Growth Period of the Elderly Population. POPULATION & ECONOMICS. 2021(06):1-21.
7. Chung KC, Shauver MJ, Birkmeyer JD. Trends in the United States in the Treatment of Distal Radial Fractures in the Elderly. *J Bone Joint Surg-Am Vol.* 2009;91A(8):1868-1873.
8. Costa ML, Achten J, Ooms A, et al. Surgical fixation with K-wires versus casting in adults with fracture of distal radius: DRAFFT2 multicentre randomised clinical trial. *BMJ-British Medical Journal.* 2022;376:7.
9. Lawson A, Naylor JM, Buchbinder R, et al. Surgical Plating vs Closed Reduction for Fractures in the Distal Radius in Older Patients A Randomized Clinical Trial. *JAMA Surg.* 2021;156(3):229-237.
10. Deune EG. A Prospective Randomized Study With No Clinically Important Differences in Closed vs Open Treatment for Distal Radius Fracture in Elderly Individuals. *JAMA Surg.* 2021;156(3):237-238.
11. Chung KVC, Kim HM, Malay S, Shauver MJ. Comparison of 24-Month Outcomes After Treatment for Distal Radius Fracture The WRIST Randomized Clinical Trial. *JAMA Netw Open.* 2021;4(6):13.
12. Hassellund SS, Williksen JH, Laane MM, et al. Cast immobilization is non-inferior to volar locking plates in relation to QuickDASH after one year in patients aged 65

years and older: a randomized controlled trial of displaced distal radius fractures. *Bone Joint J.* 2021;103B(2):247-255.

13. van Delft EAK, van Gelder TG, de Vries R, Vermeulen J, Bloemers FW. Duration of Cast Immobilization in Distal Radial Fractures: A Systematic Review. *J Wrist Surg.* 2019;8(5):430-438.
14. British Orthopaedic Association and British Society for Surgery of the Hand Blue Book Committee. Best practice for management of distal radial fractures. Published 2018. Accessed November 10, 2020. https://www.bssh.ac.uk/_userfiles/pages/files/professionals/Radius/Blue Book DRF Final Document.pdf
15. Arora R, Lutz M, Deml C, Krappinger D, Haug LZ, Gab M. A Prospective Randomized Trial Comparing Nonoperative Treatment with Volar Locking Plate Fixation for Displaced and Unstable Distal Radial Fractures in Patients Sixty-five Years of Age and Older. *J Bone Joint Surg-Am Vol.* 2011;93A(23):2146-2153.
16. Bentohami A, de Burlet K, de Korte N, van den Bekerom MPJ, Goslings JC, Schep NWL. Complications following volar locking plate fixation for distal radial fractures: a systematic review. *J Hand Surg-Eur Vol.* 2014;39(7):745-754.
17. Gong HS, Lee JO, Huh JK, Oh JH, Kim SH, Baek GH. Comparison of depressive symptoms during the early recovery period in patients with a distal radius fracture treated by volar plating and cast immobilisation. *Injury-Int J Care Inj.* 2011;42(11):1266-1270.
18. Halanski M, Noonan KJ. Cast and splint immobilization: Complications. *J Am Acad Orthop Surg.* 2008;16(1):30-40.
19. Boudissa M, Courvoisier A, Chabanas M, Tonetti J. Computer assisted surgery in preoperative planning of acetabular fracture surgery: state of the art. *Expert Rev Med Devices.* 2018;15(1):81-89.
20. Zamborsky R, Kilian M, Jacko P, Bernadic M, Hudak R. Perspectives of 3D printing technology in orthopaedic surgery. *Bratisl Med J.* 2019;120(7):498-504.
21. Dhawan A, Kennedy PM, Rizk EB, Ozbolat IT. Three-dimensional Bioprinting for Bone and Cartilage Restoration in Orthopaedic Surgery. *J Am Acad Orthop Surg.* 2019;27(5):E215-E226.
22. Kaji Y, Yamaguchi K, Nomura Y, et al. Postoperative early and proactive grip strength training program for distal radius fractures promotes earlier recovery of grip strength A retrospective study. *Medicine (Baltimore).* 2022;101(19):6.
23. Bendsoe MP, Kikuchi N. GENERATING OPTIMAL TOPOLOGIES IN STRUCTURAL DESIGN USING A HOMOGENIZATION METHOD. *Comput Meth Appl Mech Eng.* 1988;71(2):197-224.
24. Liao YC, Feng CK, Tsai MW, Chen CS, Cheng CK, Ou YC. Shape modification of the Boston brace using a finite-element method with topology optimization. *Spine.* 2007;32(26):3014-3019.
25. Mian SH, Umer U, Moiduddin K, Alkhalefah H. Finite Element Analysis of Upper Limb Splint Designs and Materials for 3D Printing. *Polymers.* 2023;15(14):25.
26. Yan W, Ding M, Kong B, Xi XB, Zhou MD. Lightweight Splint Design for Individualized Treatment of Distal Radius Fracture. *J Med Syst.* 2019;43(8):10.

27. Farina I, Singh N, Colangelo F, Luciano R, Bonazzi G, Fraternali F. High-Performance Nylon-6 Sustainable Filaments for Additive Manufacturing. *Materials.* 2019;12(23):16.

28. Toncheva A, Brison L, Dubois P, Laoutid F. Recycled Tire Rubber in Additive Manufacturing: Selective Laser Sintering for Polymer-Ground Rubber Composites. *Appl Sci-Basel.* 2021;11(18):14.

29. Ma ZL. Comparison of the effect of closed reduction small splint immobilization and cast immobilization in the treatment of distal radius fracture. *Contemporary Medical Symposium*. 2020;18(11):59-60.
