# Supplementary material for: Topology-Optimized Splints vs Casts for Distal Radius Fractures: A Randomized Clinical Trial
Source: JAMA Netw Open. 2024 Feb 2;7(2):e2354359. doi: 10.1001/jamanetworkopen.2023.54359 (PMC10837751; doi:10.1001/jamanetworkopen.2023.54359)
Supplement: Supplement 2. — eAppendix 1. Rehabilitation Exercise Program eAppendix 2. Splint and Cast Technique Methods eAppendix 3. Clinical Photos and Radiographs [file jamanetwopen-e2354359-s002.pdf]

Supplementary Online Content

Ma H, Ruan B, Li J, et al. Topology-optimized splints vs casts for distal radius fractures: a randomized clinical trial. *JAMA Netw Open.* 2024;7(2):e2354359.
doi:10.1001/jamanetworkopen.2023.54359

eAppendix 1. Rehabilitation Exercise Program
eAppendix 2. Splint and Cast Technique Methods
eAppendix 3. Clinical Photos and Radiographs

This supplementary material has been provided by the authors to give readers additional information about their work.

© 2024 Ma H et al. *JAMA Network Open.*

eAppendix 1. Rehabilitation Exercise Program

When the fracture is immobilization, active finger movement and light hand use were encouraged immediately. After 6 weeks of immobilization, the external fixation will be removed and active rehabilitation exercises should begin.

Patient exercise program following wrist fracture immobilization removal

1. Introduction

This rehabilitation program is designed for patients with distal radius fractures to provide a comprehensive and systematic set of rehabilitation guidelines after immobilization removal. This program emphasizes individual differences, which patients need to adjust according to their own circumstances.

2. Rehabilitation (HAND/WRIST/EBLOW/SHOULDER)

HAND / WRIST - Make fists

Make a fist with both hands, then relax.

Repeat this exercise 15 times, for at least 3 sessions each day (or exercise with a stress ball).

HAND / WRIST - Active Flexion/Extension

Place your arms on the table, palms together, elbows on the table slowly spread arms apart.

Place arms on chair arms with wrists hanging over, palms down.

© 2024 Ma H et al. *JAMA Network Open.*

Repeat this exercise 15 times, for at least 3 sessions each day.

HAND / WRIST - Active Radial Deviation/Ulnar Deviation

Start with your palms down and turn your palms outward with the back of your hands facing up.

Start with your palms down and turn your palms inward with the back of your hands facing up.

Repeat this exercise 15 times, for at least 3 sessions each day.

HAND / WRIST - Active Supination/Pronation

With elbows at sides, alternate turning hands palm-up and palm-down.

Repeat this exercise 10 times, for at least 3 sessions each day.

SHOULDER - Shoulder Pinch

Pull shoulders forward, backward, abduction, adduction, external rotation, and internal rotation, pinching shoulder blades together.

(Hold 5 seconds. Relax.)

Repeat this exercise 15 times, for at least 3 sessions each day.

ELBOW - Elbow AROM

Standing or siting, bend both arms to touch your shoulders, then return to your side.

Repeat this exercise 15 times, for at least 3 sessions each day.

© 2024 Ma H et al. *JAMA Network Open.*

eAppendix 2. Splint and Cast Technique Methods

Splint technique methods: In this study, we developed a mathematical model for the splint, considering its geometry, material properties, external loads, and boundary conditions. Then, we utilized a topology optimization algorithm to optimize the plywood structure based on predefined goals (e.g., maximizing stiffness, minimizing weight) and constraint conditions (e.g., material usage (polyamide, PA12), manufacturing process). By iteratively calculating, we successfully reduced unnecessary material while meeting design requirements, resulting in a lighter and more efficient plywood. To evaluate the performance of the optimized structure, we employed the finite element method for simulation and analysis. Finally, the splint body was manufactured using multi-jet fusion 3D printing technology. In order to facilitate the quick application of splints to clinical patients with distal radius fractures, we have developed 4 different sizes of prefabricated topology-optimized splints. These splints are designed to meet the majority of patient needs. However, for certain special patients, such as individuals with larger body frames or very thin individuals, the prefabricated splints may not be suitable. In such cases, we offer customized topology-optimized splints.

Cast technique methods: To begin, gather the necessary materials: cast bandage, paper cotton, and water. Start by wrapping the forearm with

© 2024 Ma H et al. *JAMA Network Open.*

paper cotton. Next, reinforce cast bandage on the dorsal or palmar skin, depending on the actual situation of fracture displacement. Finally, the cast was applied over the skin to immobilize the fracture, extending from below the elbow to the metacarpals.

© 2024 Ma H et al. *JAMA Network Open.*

eAppendix 3. Clinical Photos and Radiographs

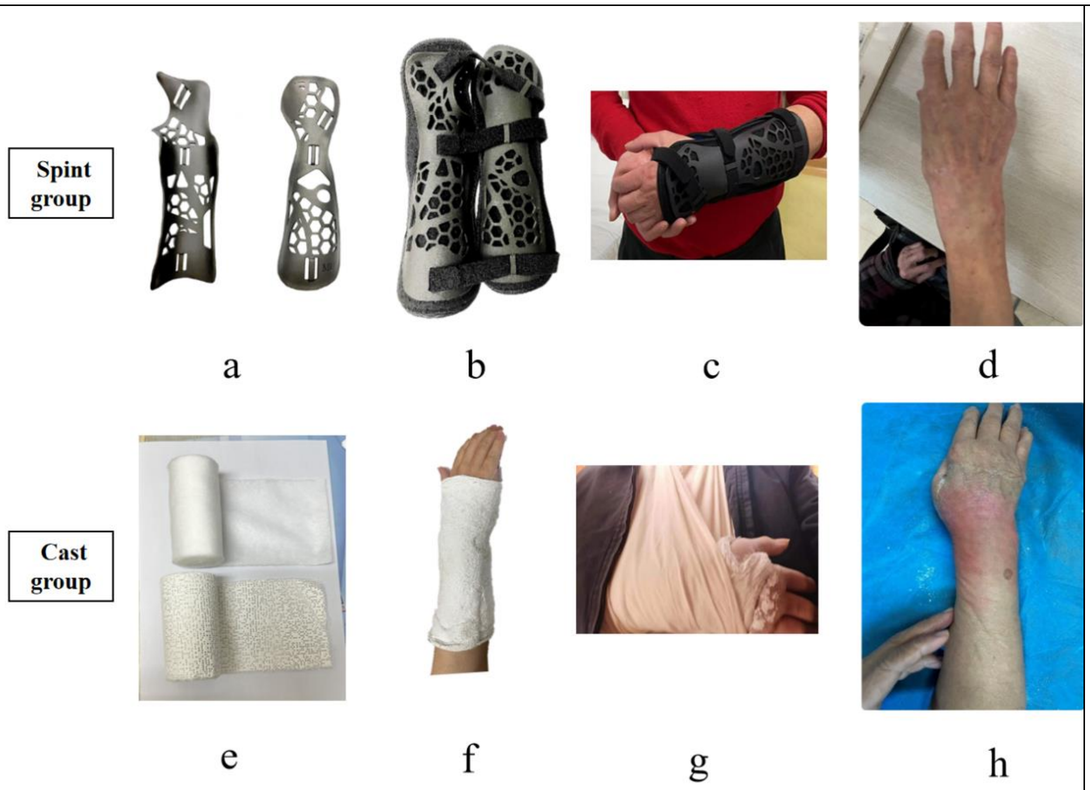

a: Model of splint   b: Solid image   c: Wearing image of patient 1

d: After removal image of patient 2

c: Material of cast   f: Model of cast immobilization

g: Wearing image of patient 3   h: After removal image of patient 4

© 2024 Ma H et al. *JAMA Network Open.*

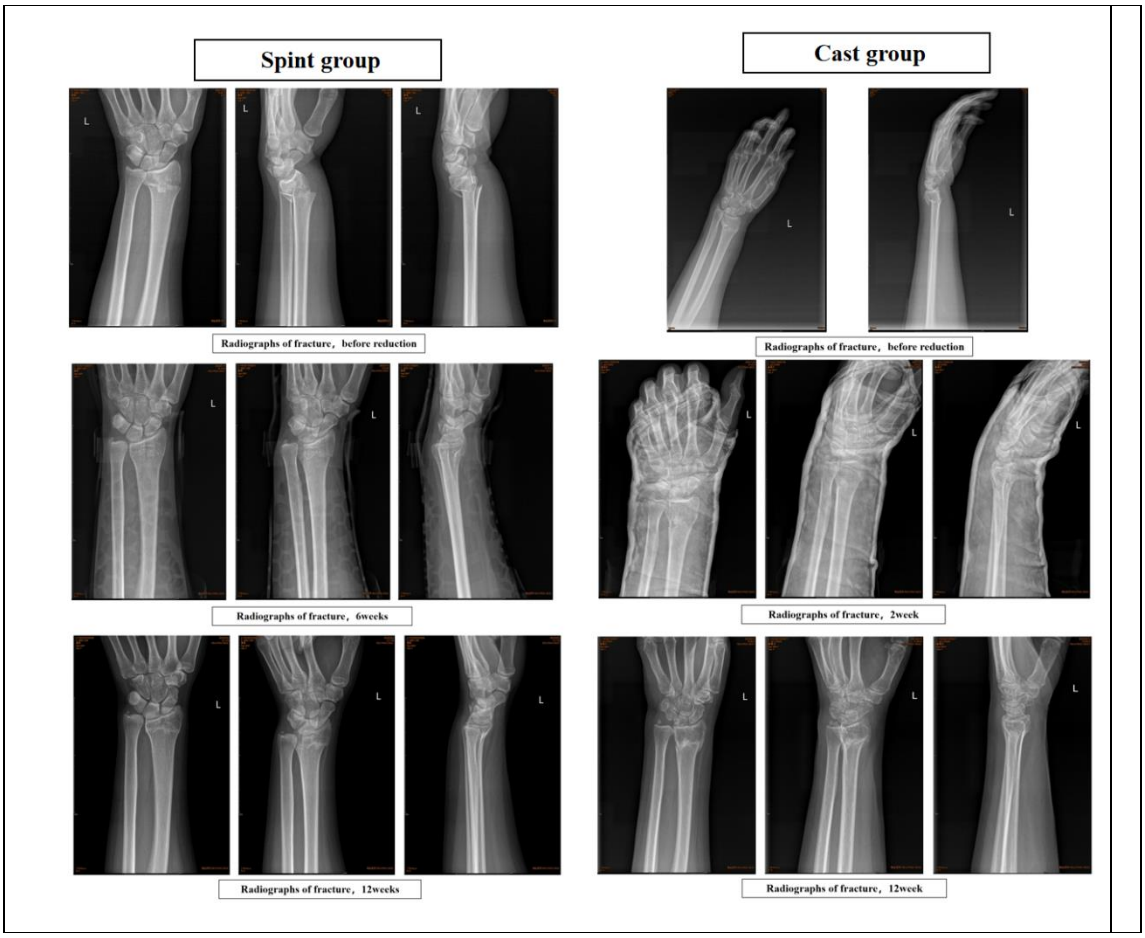

© 2024 Ma H et al. *JAMA Network Open.*
