# Supplementary material for: Topology-Optimized Splints vs Casts for Distal Radius Fractures: A Randomized Clinical Trial
Source: JAMA Netw Open. 2024 Feb 2;7(2):e2354359. doi: 10.1001/jamanetworkopen.2023.54359 (PMC10837751; doi:10.1001/jamanetworkopen.2023.54359)
Supplement: Supplement 3. — Data Sharing Statement [file jamanetwopen-e2354359-s003.pdf]

Data Sharing Statement

Ma. Topology-Optimized Splints vs Casts for Distal Radius Fractures. *JAMA Netw Open*. Published February 02, 2024. doi:10.1001/jamanetworkopen.2023.54359

Data
Data available: Yes
Data types: Deidentified participant data
How to access data: The data sets analyzed during the current study are available from the corresponding author upon reasonable request. Email:skxixiaobing@163.com
When available: With publication

Supporting Documents
Document types: Statistical/analytic code, Informed consent form
How to access documents: The statistical/analytic code and informed consent form during the current study are available from the corresponding author upon reasonable request.
skxixiaobing@163.com
When available: With publication

Additional Information
Who can access the data: Anyone requesting the data
Types of analyses: For any purpose
Mechanisms of data availability: With a signed data access agreement
Any additional restrictions: None
